# Overexposure to apoptosis via disrupted glial specification perturbs *Drosophila* macrophage function and reveals roles of the CNS during injury

**DOI:** 10.1038/s41419-020-02875-2

**Published:** 2020-08-14

**Authors:** Emma Louise Armitage, Hannah Grace Roddie, Iwan Robert Evans

**Affiliations:** Department of Infection, Immunity and Cardiovascular Disease and The Bateson Centre, University of Sheffield, Sheffield, UK

**Keywords:** Cell migration, Mechanisms of disease, Developmental biology, Cell death and immune response, Imaging the immune system

## Abstract

Apoptotic cell clearance by phagocytes is a fundamental process during development, homeostasis and the resolution of inflammation. However, the demands placed on phagocytic cells such as macrophages by this process, and the limitations these interactions impose on subsequent cellular behaviours are not yet clear. Here, we seek to understand how apoptotic cells affect macrophage function in the context of a genetically tractable *Drosophila* model in which macrophages encounter excessive amounts of apoptotic cells. Loss of the glial-specific transcription factor Repo prevents glia from contributing to apoptotic cell clearance in the developing embryo. We show that this leads to the challenge of macrophages with large numbers of apoptotic cells in vivo. As a consequence, macrophages become highly vacuolated with cleared apoptotic cells, and their developmental dispersal and migration is perturbed. We also show that the requirement to deal with excess apoptosis caused by a loss of *repo* function leads to impaired inflammatory responses to injury. However, in contrast to migratory phenotypes, defects in wound responses cannot be rescued by preventing apoptosis from occurring within a *repo* mutant background. In investigating the underlying cause of these impaired inflammatory responses, we demonstrate that wound-induced calcium waves propagate into surrounding tissues, including neurons and glia of the ventral nerve cord, which exhibit striking calcium waves on wounding, revealing a previously unanticipated contribution of these cells during responses to injury. Taken together, these results demonstrate important insights into macrophage biology and how *repo* mutants can be used to study macrophage–apoptotic cell interactions in the fly embryo. Furthermore, this work shows how these multipurpose cells can be ‘overtasked’ to the detriment of their other functions, alongside providing new insights into which cells govern macrophage responses to injury in vivo.

## Introduction

Understanding the interactions between macrophages and apoptotic cells is an important biological question: failures in how immune cells deal with apoptotic cell death can lead to damaging autoimmune conditions^1^. Apoptotic cell clearance also plays a critical role in the resolution of inflammation and in the reprogramming of immune cells during this process^2^. Furthermore, interactions between dying cells and macrophages occur at numerous sites of pathology, including at sites of atherosclerosis^3^ and in the chronically inflamed lungs of patients with chronic obstructive pulmonary disease (COPD)^4^. As such, macrophage–apoptotic cell interactions have the potential to impact these diseases and many other damaging human conditions^1^.

*Drosophila* has proven an excellent organism with which to study innate immunity^5^, haematopoiesis^6^ and blood cell function^7^. *Drosophila* blood is dominated by macrophage-like cells (plasmatocytes), with these cells making up 95% of the blood cells (hemocytes) in the developing embryo^8^. These embryonic macrophages disperse over the entire embryo during development, phagocytosing apoptotic cells and secreting matrix as they migrate^7^. Alongside their functional and morphological similarities to vertebrate macrophages, *Drosophila* macrophages are specified through the action of related transcription factors to those used in vertebrate haematopoiesis^9^. Failed dispersal or ablation of these embryonic macrophages leads to developmental abnormalities and failure to hatch to larval stages^10–12^. *Drosophila* macrophages are also able to respond to injury, mounting inflammatory responses to epithelial wounds^13^. In embryos and pupae, wounding elicits a rapid calcium wave through the epithelium, a process that requires transient receptor potential (Trp) channel function^14,15^. The increase in cytoplasmic calcium drives activation of dual oxidase (DUOX) and production of hydrogen peroxide, which is necessary for immune cell recruitment^14^, resembling events upon tissue damage in higher organisms such as zebrafish^16,17^.

In addition to macrophages, the other main phagocyte population within *Drosophila* embryos is the glia of the developing nervous system^18^. *Drosophila* embryonic macrophages interact with the developing ventral nerve cord (VNC) and the glial cells that encase it, as they disperse along the ventral side of the embryo^19^. The VNC contains two populations of glia—the Sim-positive midline glia that help establish the ladder-like structure of neurons early in development and Repo-positive lateral glia^20^. Repo is a homeodomain transcription factor that specifies glial fate, though defects in *repo* mutants are not obvious until late in embryonic development, when a decrease in the numbers of glial cells becomes apparent^21–23^. While early glial markers are not lost in *repo* mutants^22,24^, Repo is required for the expression of at least some phagocytic receptors used by these cells^25^. Despite an overlap in the transcription factors used to specify blood cells and glia (e.g., Gcm and Gcm2)^26,27^, these cells are derived from distinct progenitors and Repo antagonises haematopoiesis to promote a glial fate^28^. Nonetheless, both macrophages and *repo*-positive glial cells express a similar repertoire of receptors for apoptotic cells, including Draper and Simu^29^. The close interactions between these phagocytes means that, should one population fail to clear apoptotic cells, it is likely that the other population would be able to both detect this deficiency and be able to compensate. Loss of apoptotic cell receptors such as Simu leads to a buildup of apoptotic cells within developing embryos^30^ and this impairs macrophage behaviours, including their developmental dispersal and inflammatory responses^31^. However, whilst mutants such as *simu* enable exposure of macrophages to elevated numbers of apoptotic cells in vivo, this approach is not ideal, as it also perturbs receptors that may be involved in immune cell reprogramming^32,33^.

To investigate interactions between macrophages and apoptotic cells in more detail, we stimulated macrophages with enhanced levels of apoptotic cells using a genetic approach that did not alter the macrophages themselves. By impairing glial differentiation using *repo* mutants, we increased the number of apoptotic cells macrophages face within the developing embryo. This enhanced apoptotic challenge impaired macrophage dispersal, migration and their inflammatory responses to wounds. In this background, clearance of apoptotic cells by macrophages is not perturbed, and migration can be rescued by preventing apoptosis. Surprisingly, and in contrast to phagocytic receptor mutants, blocking apoptotic cell death in the presence of defective glia failed to rescue wound responses. Further analysis revealed that injury-induced calcium waves propagate beyond the wounded epithelium, and that this process is defective in *repo* mutants. This suggests that glial cells play an active role in the propagation of even the earliest responses to wounding. Thus, this model provides a unique insight into how macrophage–apoptotic cell interactions dictate macrophage responses to injury and the cell types that contribute to the activation of those responses.

## Materials and methods

### Fly lines and husbandry

*Drosophila melanogaster* fruit flies were reared at 25 °C on cornmeal/agar/molasses media (see Supplementary Table 1 for recipe). Depending on the exact alleles and transgenes needed for each experiment, either *srp-GAL4*^34^ and/or *crq-GAL4*^13^ were used interchangeably to label *Drosophila* macrophages in the embryo in combination with *UAS-GFP* and/or *UAS-red stinger*^35^. *e22c-GAL4* (VNC and epithelium)^36^, *act5C-GAL4* (ubiquitous)^37^, *da-GAL4* (ubiquitous)^38^, *elav-GAL4* (neuronal)^39^ and *repo-GAL4* (glial cells)^40^ were used to drive expression in other tissues. *UAS-GCaMP6M*^41^ was used to image changes in cytoplasmic calcium concentration. Experiments were conducted on a *w*^*1118*^ background, and the following mutant alleles were used: *Df(3L)H99*^42^, *repo*^03702^ (see refs. ^21–23^), *simu*^*2*^ (see ref. ^30^). See Supplementary Table 2 for a full list of genotypes used in this study and sources of the *Drosophila* lines used. Embryos were collected from apple juice agar plates on which flies had laid overnight at 22 °C. Embryos were washed off plates with distilled water and dechorionated in bleach for 1–2 min. Bleach was thoroughly washed away with distilled water ahead of fixation or mounting of embryos for live imaging. The absence of the fluorescent balancers *CTG*, *CyO dfd*, *TTG* and *TM6b dfd*^43,44^ was used to select homozygous mutant embryos after dechorionation.

### Fixation and immunostaining

Dechorionated embryos were fixed and stained as per Roddie et al.^31^. Antibodies were diluted in PATx (0.1% Triton-X100 (Sigma-Aldrich), 1% BSA (Sigma-Aldrich) in PBS (Oxoid, Thermo Fisher, MA, USA)). Rabbit anti-GFP (ab290 1:1000; Abcam, Cambridge, UK) or mouse anti-GFP (ab1218 1:200; Abcam) were used to detect GFP-labelled macrophages. Rabbit anti-cDCP-1 (9578S 1:1000; Cell Signaling Technologies), mouse anti-Repo (concentrate of clone 8D12 used at 1:1000; Developmental Studies Hybridoma Bank, University of Iowa, USA) or mouse anti-Futch (supernatant of clone 22C10 used at 1:200; Developmental Studies Hybridoma Bank) were also used as primary antibodies. Goat anti-mouse or goat anti-rabbit secondary antibodies conjugated to AlexaFluor568, AlexaFluor488 (A11036 and A11034; Invitrogen, Thermo Fisher) or FITC (115-095-146; Jackson Immunoresearch, Cambridge, UK) were used to detect primary antibodies; these were diluted from stock solutions made according to the recommendations of the supplier (1:400 in PATx). Stained embryos were stored in DABCO mountant (Sigma-Aldrich) and mounted on slides for imaging.

### Imaging and analysis

Immunostained embryos were imaged using a ×40 objective lens (CFI Super Plan Fluor ELWD 40x, NA 0.6) on a Nikon A1 confocal. In order to quantify apoptotic cell clearance, embryos containing GFP-labelled macrophages were stained for GFP and cDCP-1. Only those macrophages fully in view on the ventral midline and within a 10-μm deep sub-stack that corresponds to the region between the epidermis and VNC on the ventral midline were analysed. The number of cDCP-1 punctae within macrophage, per embryo (phagocytic index) was calculated from slices in the 10-μm deep sub-stack to provide a measure of apoptotic cell clearance. Sub-stacks were blinded before analysis, and a minimum of four macrophages were counted per embryo. To quantify vacuolation, the number of vacuoles within a single z-slice at which each macrophage exhibited its maximal cross-sectional area was scored. Vacuoles were discriminated via their exclusion of cytoplasmic GFP. Only macrophages on the midline between the epidermis and VNC and fully within view were analysed.

Developmental dispersal was assessed by counting numbers of segments lacking GFP-labelled macrophages on the ventral side of the VNC in stage 13/14 embryos. Embryos that had been fixed and stained for GFP were scored using a Leica MZ205 FA fluorescent dissection microscope with a PLANAPO ×2 objective lens. The same embryos were orientated ventral-side-up, imaged on a Nikon A1 confocal, and numbers of macrophages between epithelium and VNC counted (segments 4–8 were scored).

For live imaging of macrophage morphology, migration speed and wound responses and calcium dynamics upon injury, live embryos were mounted in voltalef oil (VWR) as per Evans et al.^19^ and imaged on a Perkin Elmer UltraView Spinning Disk system using a ×40 objective lens (UplanSApo ×40 oil, NA 1.3). Embryos that were not ventral-side-up, that rolled during imaging or with large leaks post wounding were excluded from subsequent analyses. Epithelial wounds were made on the ventral side of embryos using a nitrogen-pumped Micropoint ablation laser (Andor, Belfast, UK), as per Evans et al.^45^. Macrophage inflammatory responses to wounds were imaged and analysed as per Roddie et al.^31^. Macrophage wound responses (numbers of macrophage at or touching the wound edge at 60 min divided by wound area in μm^2^) were normalised to control levels. The percentage of macrophages responding (% responders) was calculated by counting the proportion of macrophages present immediately following wounding that reached the wound within 60 min; macrophages already at the wound or absent from the field of view immediately after wounding were not considered in this analysis. Numbers of macrophages in the image taken prior to wounding (pre-wound) were counted as a measure of macrophages available to respond to the injury. The percentage of macrophages leaving the wound (% leavers) is the proportion of macrophages present at the wound site at any point during a 60-min movie that leave the wound site; macrophages retaining contacts with the wound site or edge were not scored as leavers.

For analysis of random migration, 60-min movies of GFP and red stinger double-labelled macrophages were made with macrophage position imaged every 2 min on the ventral midline between the overlying epithelium and VNC. Image stacks were despeckled, and the movements of macrophages within maximum projections tracked as per Roddie et al.^31^. The manual tracking and Ibidi chemotaxis plugins were used to calculate speed per macrophage, per embryo, in Fiji^46^.

Calcium dynamics were imaged using GCaMP6M expressed using a range of GAL4 lines. Calcium responses were quantified from average projections of z-stacks collected immediately before and after wounding. Initial wound responses of projections corresponding to epithelial z-slices (from *da-GAL4,UAS-GCaMP6M* embryos) were quantified by measuring the area of the GCaMP6M response and the corresponding mean gray value (MGV) of this region of interest (ROI) in Fiji immediately after wounding (F1). The same ROI was then used to measure the MGV of GCaMP6M fluorescence in the pre-wound image (F0). Both the area of the initial response and the F1/F0 ratio of MGVs were used as measures of the calcium response to injury (Fig. 7c, d). For analysis of glial responses and the cells within the VNC more broadly (neurons and glia), *repo-GAL4* and *e22c-GAL4* were used to drive GCaMP6M expression, respectively. Responding glial cells were manually selected using the freehand selection tool in Fiji (Fig. 8h, i), while the outer edge of the vitelline membrane was used to define a ROI to quantify GCaMP6M intensity for projections constructed from deeper volumes of the z-stack (Fig. 8c).

### Image processing and statistical analyses

Images were despeckled in Fiji before maximum or average projections were made. All projections or z-stacks to be analysed were blinded ahead of quantification, and Adobe Photoshop was used to assemble figures.

Data are displayed on scattergraphs with lines representing the mean and with standard deviation used as a measure of spread (shown via error bars). Two-sided statistical analyses were performed in GraphPad Prism. Data were not assumed to be normally distributed, and Mann–Whitney and Kruskall–Wallis tests were used. A Pearson test was used to test for correlations between numbers of vacuoles and cell speed (Fig. 4f, g). Details of statistical analyses, post-tests used to correct for multiple comparisons, *P* values and sample sizes (number of embryos) are reported in figure legends. Experiments were repeated in at least triplicate with embryos taken from laying cages containing greater than 50 adult flies of the same genotype. Minimum sample sizes were estimated based on numbers of embryos sufficient to see a difference of 20% from previous studies. For live-imaging experiments, mutant or control embryos of the correct developmental stage were selected at random from pools of dechorionated embryos of the same genotype and mounted on the same microscope slide for each replicate.

Immunostained embryos are representative examples taken from batches of pooled embryos collected across multiple days. Control and mutant embryos were stained at the same time using master mixes of reagents that was split to stain the different genotypes.

## Results

### Embryonic macrophages disperse in close contact with the developing central nervous system

**Fig. 1:**
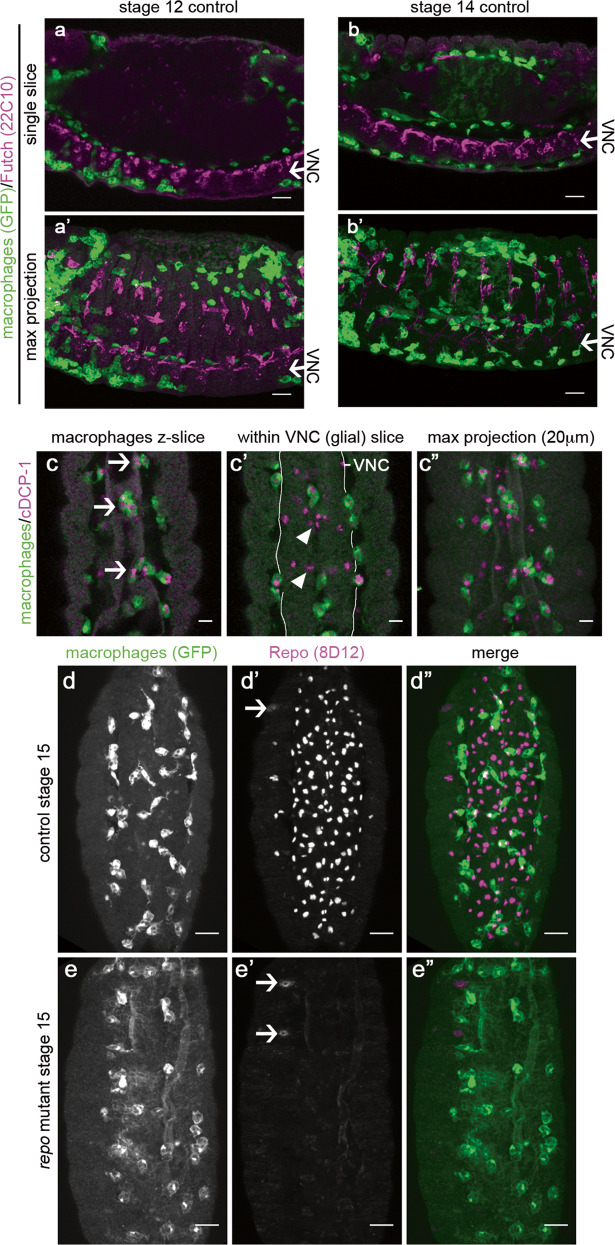
Interaction of macrophages and glial cells during *Drosophila* embryonic development. **a**, **b** Single z-slices (**a**, **b**) and maximum projections (**a’**, **b’**) of immunostained control embryos with GFP-labelled macrophages (green) showing progression of macrophages along both sides of the ventral nerve cord (VNC, Futch staining; magenta) at stage 12 (**a**) and 14 (**b**). Arrows indicate the position of VNC; embryos are laterally orientated with anterior to the left, and ventral down. **c** Ventral views of a stage 15 control embryo immunostained for apoptotic cells (anti-cDCP-1, magenta) and macrophages (anti-GFP, green); panels show single z-slices showing engulfment of apoptotic cells by macrophages (arrows, **c**) and apoptotic cells within the VNC (arrowheads, **c’**), and a maximum projection of this region corresponding to a 20-μm deep z-stack (**c”**); a white line in (**c’**) indicates the edges of the VNC—macrophages within these lines are sitting within the midline pores that span this structure. **d**, **e** Ventral views of stage 15 control and *repo* mutant embryos immunostained for GFP to show macrophages (**d**, **e**) and anti-Repo (**d’**, **e’**); macrophages and Repo are green and magenta, respectively, in merged images (**d”**, **e”**); arrows in anti-Repo channel indicate non-nuclear staining likely to be cross-reactivity to another epitope. All scale bars represent 10 μm. Genotypes are as follows: *w;srp-GAL4,UAS-GFP/* + *;crq-GAL4,UAS-GFP/* + (**a**, **b**), *w;;crq-GAL4,UAS-GFP* (**c**, **d**), *w;;repo*^*03702*^*,crq-GAL4,UAS-GFP* (**e**).

*Drosophila* embryonic macrophages migrate out from the presumptive head region to disperse over the entire embryo during development^7^. Migration along the developing ventral nerve cord (VNC) is an essential route for macrophage dispersal, with macrophages contacting the overlying epithelium and glial cells on the surface of the nerve cord (Fig. 1a, b). During dispersal, macrophages encounter and clear apoptotic cells (Fig. 1c), while VNC glia also phagocytose dying cells^47^. The interaction of macrophages and glia suggested to us that impairing glial-mediated apoptotic cell clearance could increase exposure of macrophages to apoptotic cell death in vivo, thus providing a model with which apoptotic cell–macrophage interactions and their effects on macrophage behaviour could be studied in detail.

### Increased efferocytosis by embryonic macrophages in the absence of functional glial cells

Macrophages and glia use the same phagocytic receptors to clear apoptotic cells^30,48,49^, and the absence of these receptors perturbs efferocytosis, in turn disrupting macrophage function^31,45^. While this suggests that apoptotic cells modulate macrophage behaviour in flies, these interventions remove genes that also control macrophage fate^32^. Therefore, in order to expose macrophages to increased amounts of apoptotic cell death without directly impacting their own specification mechanisms or removing important regulators of phagocytosis, we targeted the glia of the VNC. We hypothesised that blocking glial-mediated clearance would lead to macrophages becoming exposed to increased numbers of apoptotic cells due to the inability of glial cells to contribute to this process.

The homeodomain transcription factor Repo is expressed by all glial cells within the developing VNC^21–23^, except the midline glia, which are specified via the action of *sim*^50^. Repo is absolutely required for normal specification of these glial cells, and absence of *repo* leads to the failure to express a variety of phagocytic receptors required for apoptotic cell clearance^25^. Repo is not expressed by macrophages in the developing embryo (Fig. 1d; Lee and Jones^40^), and *repo*^*03702*^ mutants lack detectable protein expression in the VNC (Fig. 1e). Consistent with the failed condensation of the VNC in the absence of *repo* function^22^, there is a small but statistically significant increase in the length of body segments at stage 15 in *repo* mutants compared with controls (Supplementary Fig. 1a, b). As per other VNC-specific defects^21,22^, this phenotype cannot be detected at earlier stages of development (Supplementary Fig. 1c).

**Fig. 2:**
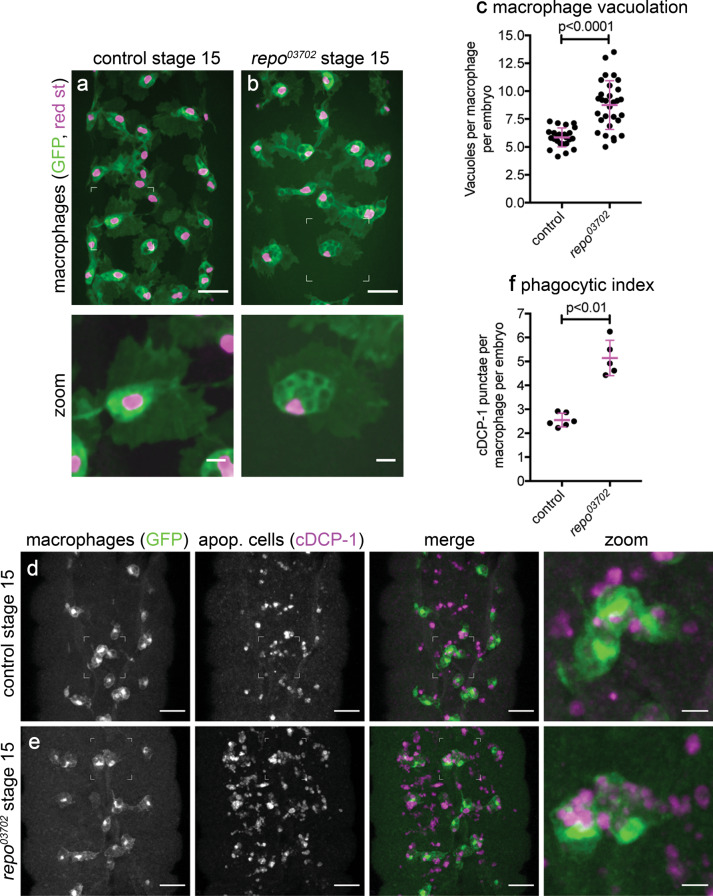
Increased macrophage-mediated apoptotic cell clearance in the absence of glial specification. **a**, **b** Ventral views of stage 15 control (**a**) and *repo* mutant (**b**) embryos containing GFP and red stinger-expressing macrophages; lower panels show zooms of macrophages indicated by boxes in upper panels. **c** Scattergraph of vacuoles per macrophage per embryo from genotypes in (**a**, **b**); *P* < 0.0001 via Mann–Whitney test (*n* = 24 and 30). **d**, **e** Ventral views of stage 15 control (**d**) and *repo* mutant (**e**) embryos containing GFP-labelled macrophages immunostained for GFP (green in merge) and apoptotic cells (anti-cDCP-1, magenta in merge); zooms show macrophages indicated in boxed regions. **f** Scattergraphs of phagocytic index (cDCP-1 puncta per macrophage per embryo); *P* < 0.0001 (*n* = 101 and 69 cells from 6 and 5 embryos, respectively) and *P* = 0.0043 (*n* = 6 and 5 embryos, respectively) via Mann–Whitney tests. Scale bars represent 20 μm or 5 μm in zooms; error bars and lines show standard deviation and mean, respectively (**c**, **f**); genotypes are *w;srp-GAL4,UAS-GFP/srp-GAL4,UAS-red stinger* (**a**, **c**), *w;srp-GAL4,UAS-GFP/srp-GAL4,UAS-red stinger;repo*^*03702*^ (**b**, **c**), *w;;crq-GAL4,UAS-GFP* (**d**, **f**) and *w;;repo*^*03702*^*,crq-GAL4,UAS-GFP* (**e**, **f**).

To test whether failed glial specification would lead to macrophages encountering increased numbers of apoptotic cells in the developing embryo, we analysed macrophage morphology. Macrophages in *repo* mutants are highly vacuolated compared with controls (Fig. 2a–c); vacuoles within *Drosophila* embryonic macrophages typically contain previously engulfed apoptotic cells^51^. To test whether apoptotic cell clearance by macrophages is increased in *repo* mutants, control and *repo* mutant embryos were immunostained for a marker of apoptotic cell death (cleaved DCP-1 (cDCP-1) immunostaining; DCP-1 is cleaved by caspases during apoptosis)^52^. Macrophages in *repo* mutants contain far higher numbers of cDCP-1-positive inclusions compared with controls (Fig. 2d–f). Furthermore, macrophages can be labelled using *crq-GAL4* or *srp-GAL4* in a *repo* mutant background and efficiently engulf apoptotic cells (Fig. 2), consistent with their normal specification and physiology. There is no difference in the overall numbers of macrophages within embryos in *repo* mutants compared with controls (Supplementary Fig. 2), suggesting that macrophage proliferation is unaffected by loss of *repo* function, and that increased levels of apoptotic cell clearance do not lead to macrophage cell death. Taken together, these results suggest that loss of *repo* function is a suitable tool via which the effects of increased macrophage–apoptotic cell contact can be analysed in vivo.

### An increased burden of apoptotic cell clearance is associated with impaired developmental dispersal of macrophages

**Fig. 3:**
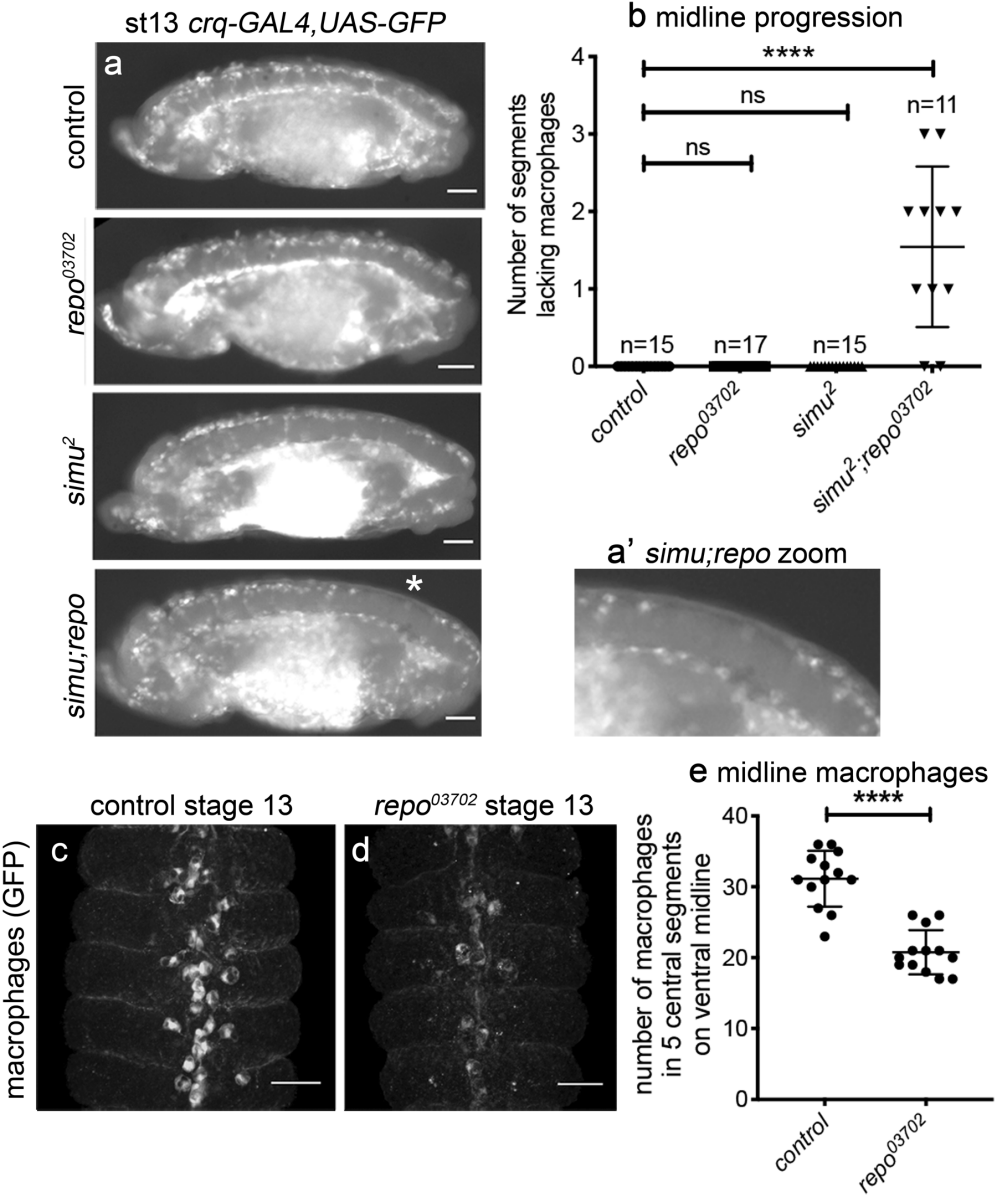
Excessive amounts of apoptotic cell death impair macrophage dispersal. **a** Lateral views of stage 13/14 control, *repo*, *simu* and *simu;repo* double-mutant embryos containing GFP-labelled macrophages. A complete line of macrophages is present on midline on the ventral side of the VNC in all genotypes, with the exception of *simu;repo* double mutants (gap indicated via an asterisk); **a’** shows zoom of this region. **b** Scattergraph showing quantification of midline progression defects (numbers of segments lacking macrophages on the ventral side of the VNC); *P* < 0.0001 (for *simu;repo* vs each other genotype; no other comparisons are significantly different) via a Kruskall–Wallis test with a Dunn’s multiple comparaison post-test; *n* = 15 (controls), 17 (*repo* mutants), 15 (*simu* mutants) and 11 (*simu;repo*). **c**, **d** Ventral views of stage 13 control and *repo* mutant embryos containing GFP-expressing macrophages (immunostained via anti-GFP). **e** Scattergraph showing quantification of numbers of macrophages in five central segments on the ventral midline; *P* < 0.0001 via Mann–Whitney test, *n* = 13 (controls), 13 (*repo* mutants). Scale bars represent 50 μm (**a**) and 25 μm (**c**, **d**); lines and error bars on scattergraphs show mean and standard deviation, respectively; **** indicates *P* < 0.0001. Genotypes are *w*^*1118*^*;;crq-GAL4,UAS-GFP* (control), *w*^*1118*^*;;P{PZ}repo*^*03702*^*,crq-GAL4,UAS-GFP* (*repo*^*03702*^), *w*^*1118*^*;simu*^*2*^*;crq-GAL4,UAS-GFP* (*simu*^*2*^) and *w*^*1118*^*;simu*^*2*^*;P{PZ}repo*^*03702*^*,crq-GAL4,UAS-GFP* (*simu;repo*).

Apoptotic cells represent the top priority for *Drosophila* macrophages to respond to within developing embryos^53^. As a result, increased numbers of apoptotic cells have the potential to disrupt macrophage behaviours, such as their dispersal and recruitment to sites of tissue injury. *repo* mutants lack gross dispersal defects, with macrophages present along both sides of the VNC at stage 13 (Fig. 3a, b). However, reduced numbers of macrophages are present on the ventral midline in *repo* mutants (Fig. 3c–e), suggesting an impairment in dispersal, especially since there is no decrease in total numbers of macrophages within *repo* mutant embryos (Supplementary Fig. 2). Further increasing apoptotic burden in a *repo* mutant background (via removal of the apoptotic cell clearance receptor Simu) causes large dispersal defects (Fig. 3a, b), comparable to those observed when critical regulators of migration are absent, e.g., SCAR/WAVE^51^. Combined, these results indicate that challenge of macrophages with excessive amounts of apoptosis can impair developmental dispersal of macrophages.

### Apoptotic cells are responsible for attenuation of macrophage migration in *repo* mutants

**Fig. 4:**
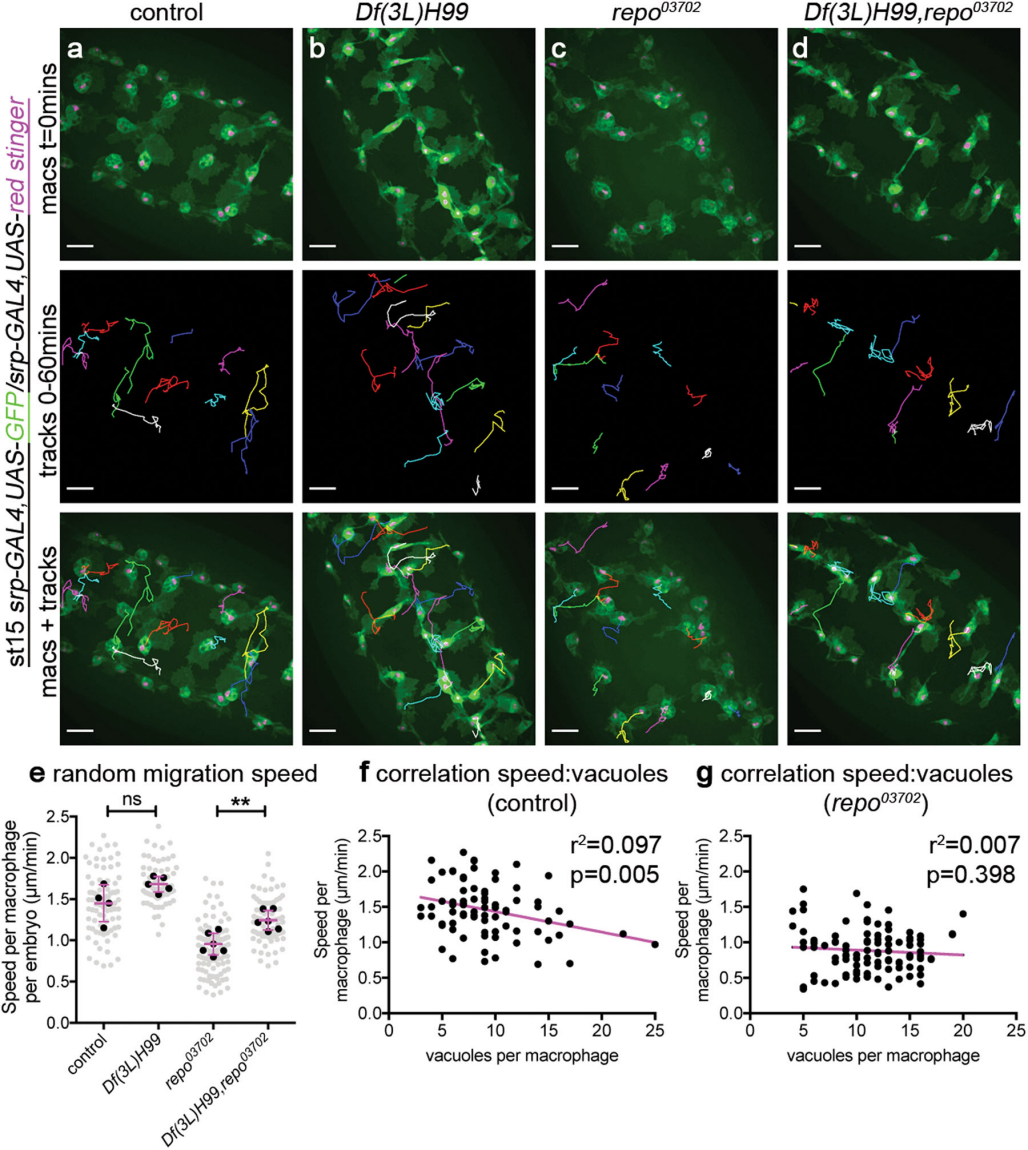
Excessive amounts of apoptotic cells impair macrophage migration. **a**–**d** Ventral images of GFP and red stinger-labelled macrophages (green and magenta, respectively) in stage 15 control (**a**), *Df(3L)H99* mutant (**b**), *repo*^*03702*^ mutant (**c**) and *Df(3L)H99,repo*^*03702*^ double-mutant embryos (**d**). Upper panels show initial position of macrophages on the ventral midline; central panels show tracks taken from subsequent 60-min period from the timepoint shown in the upper panel; the lower panel shows overlay of tracks over initial positions of macrophages. **e** Superplot showing speed per macrophage per embryo (black dots) in μm per min; grey dots represent individual cell speeds to show range of migration speeds in each genotype. Lines and error bars show mean and standard deviation, respectively (embryo averages). Removing apoptosis from a *repo* mutant background (*Df(3L)H99,repo*) rescues migration speed (*P* = 0.0095 via Mann–Whitney test; *n* = 4, 5, 6 and 6 embryos (left to right)). **f**, **g** Scatterplots of macrophage speed and vacuolation for macrophages in control (**f**) and *repo* mutant (**g**) embryos (80 and 103 macrophages analysed from 6 and 10 control and *repo* mutant embryos, respectively). Linear regression lines shown in magenta. Scale bars represent 20 μm; ns and ** indicate not significant and *P* < 0.01, respectively. Genotypes are *w*^*1118*^*;srp-GAL4,UAS-red stinger/srp-GAL4,UAS-GFP* (control), *w*^*1118*^*;srp-GAL4,UAS-red stinger/srp-GAL4,UAS-GFP;Df(3L)H99* (*Df(3L)H99*), *w*^*1118*^*;srp-GAL4,UAS-red stinger/srp-GAL4,UAS-GFP;P{PZ}repo*^*03702*^ (*repo*^*03702*^) and *w*^*1118*^*;srp-GAL4,UAS-red stinger/srp-GAL4,UAS-GFP;Df(3L)H99,P{PZ}repo*^*03702*^ (*Df(3L)H99,repo*^*03702*^).

To test the effects of increased apoptotic cell exposure on macrophage migration, we tracked the movements of macrophages on the ventral midline at stage 15 after completion of developmental dispersal, a point at which macrophages exhibit wandering/'random migration'. In *repo* mutant embryos, macrophages move at significantly slower speeds compared with those in controls (Fig. 4a–e). In order to test whether this attenuation of migration speed is dependent on interactions with apoptotic cells, we removed all developmental apoptosis from a *repo* mutant background using the *Df(3L)H99* deficiency, which deletes the pro-apoptotic genes *Hid*, *reaper* and *grim*^42^. In these *repo* mutants that lack apoptosis, there is a significant rescue of macrophage migration speed (Fig. 4c–e), suggesting that it is interactions with apoptotic cells that impair macrophage migration in vivo, rather than defective glial specification per se. There is no correlation between the degree of macrophage vacuolation and migration speed in *repo* mutants, and only a small correlation in control embryos (Fig. 4f, g). Therefore, engulfment of apoptotic cells is not required to decrease macrophage migration speeds in vivo.

### Functional glia are required for normal macrophage migration to wounds

**Fig. 5:**
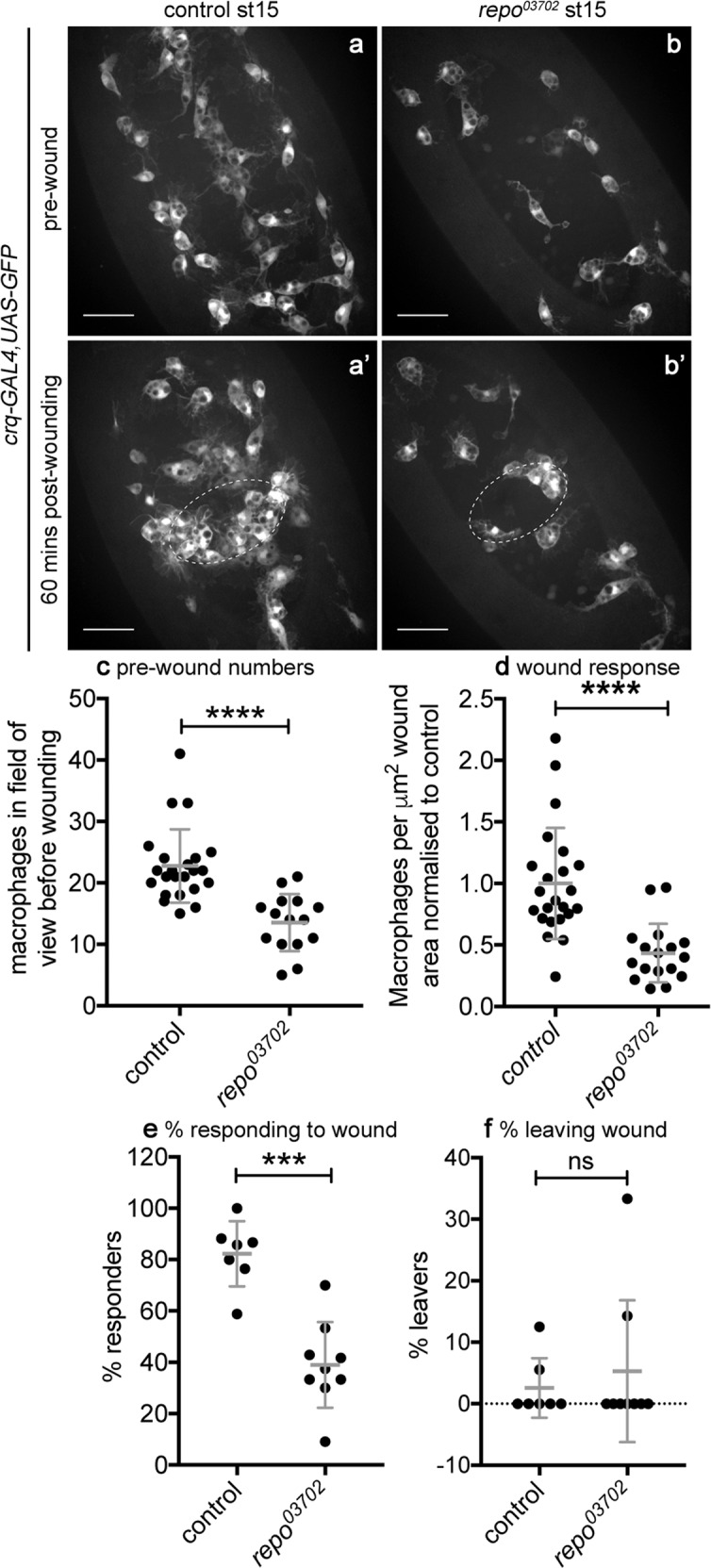
*repo* is required for normal macrophage inflammatory responses to wounding. **a, b** Ventral views showing localisation of GFP-labelled macrophages on the ventral midline in control (**a**, **a’**) and *repo* mutant (**b**, **b’**) embryos immediately before wounding (**a**, **b**) and at 60-min post wounding (**a’**, **b’**); white-dotted ellipses indicate wound edges. **c** Scattergraph showing numbers of macrophages in the field of view ahead of wounding per embryo; *P* < 0.0001 via Mann–Whitney test (*n* = 23 and 15 control and *repo*, respectively). **d** Scattergraph showing wound responses quantified via density of macrophages at wounds, normalised to control; *P* < 0.0001 via Mann–Whitney test (*n* = 23 and 17 control and *repo*, respectively). **e** Scattergraph showing percentage of macrophages responding to wounds; *P* = 0.0003 via Mann–Whitney test (*n* = 7 and 9 control and *repo*, respectively). **f** Scattergraph showing percentage of macrophages that leave the wound (having initially migrated to the wound); *P* > 0.999 via Mann–Whitney test (*n* = 7 and 9 control and *repo*, respectively). Lines and error bars in scattergraphs show mean and standard deviation, respectively; scale bars represent 20 μm; ns, *** and **** indicate not significant (*P* > 0.05), *P* < 0.001 and *P* < 0.0001, respectively. Genotypes are *w;;crq-GAL4,UAS-GFP* (control) and *w;crq-GAL4,UAS-GFP,repo*^*03702*^ (*repo*^*03702*^).

**Fig. 6:**
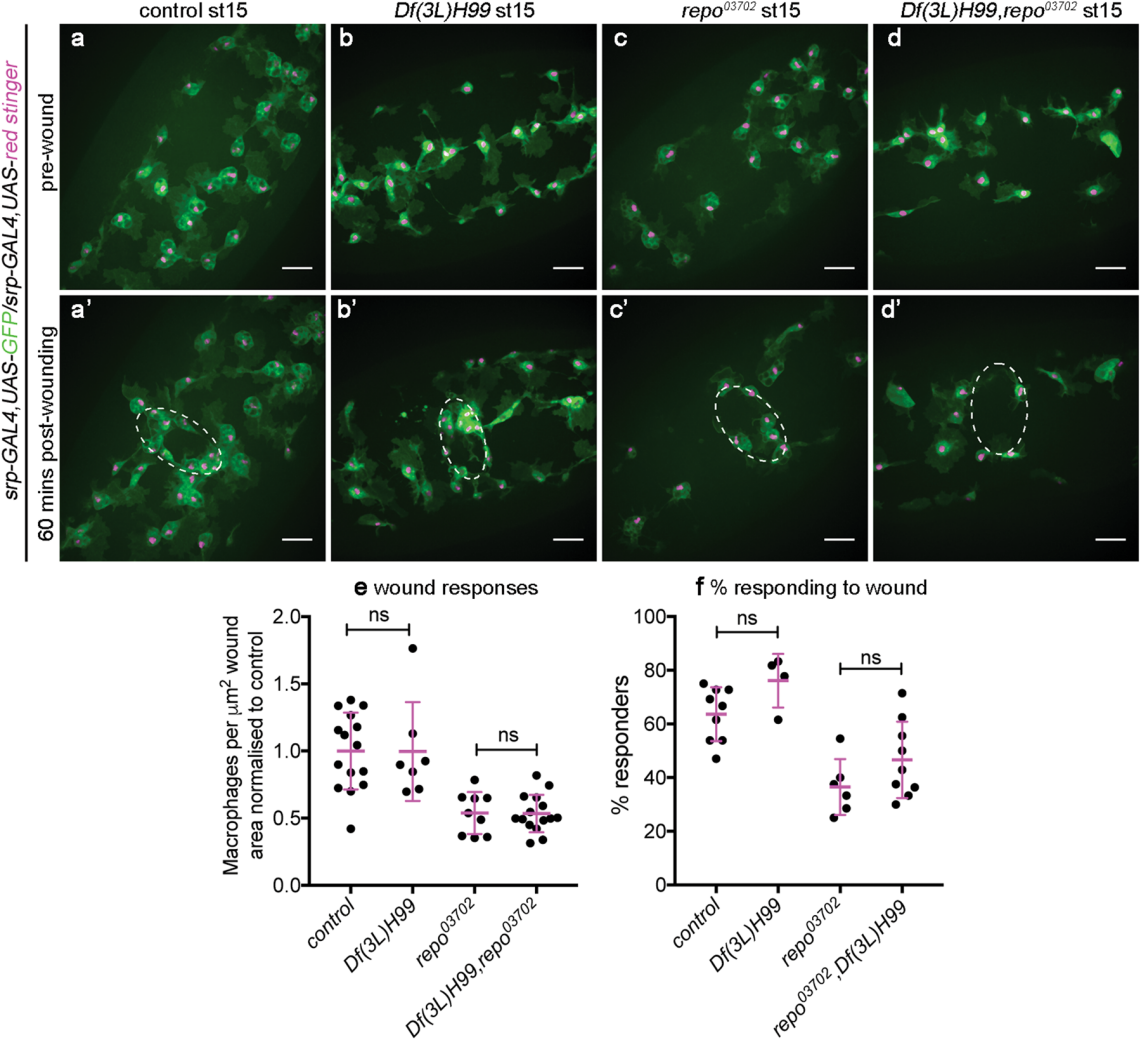
Loss of *repo* impairs wound responses, but contact with apoptotic cells is not the overriding cause of this defect. **a**–**d** Ventral views showing localisation of GFP and red stinger-labelled macrophages on the ventral midline in control (**a**, **a’**), *Df(3L)H99* (**b**, **b’**), *repo* (**c**, **c’**) and *Df(3L)H99,repo* double-mutant embryos (**d**, **d’**) immediately before wounding (**a**–**d**) and at 60 min post wounding (**a’**–**d’**); white-dotted ellipses indicate wound edges. **e** Scattergraph showing wound responses quantified via density of macrophages at wounds, normalised to control; Mann–Whitney tests used to compare control vs *Df(3L)H99* (*n* = 15 and 7, respectively; *P* = 0.63) and *repo* vs *Df(3* *L)H99,repo* (*n* = 9 and 15, respectively; *P* > 0.99). **f** Scattergraph showing percentage of macrophages responding to wounds; Mann–Whitney tests used to compare control vs *Df(3L)H99* (*n* = 9 and 4, respectively *P* = 0.055) and *repo* vs *Df(3L)H99,repo* (*n* = 6 and 6, respectively; *P* = 0.17). Lines and error bars in scattergraphs show mean and standard deviation, respectively; scale bars represent 20 μm; ns indicates not significant (*P* > 0.05). Genotypes are *w;srp-GAL4,UAS-GFP/srp-GAL4,UAS-red stinger* (control), *w;srp-GAL4,UAS-GFP/srp-GAL4,UAS-red stinger;Df(3L)H99* (*Df(3L)H99*), *w;srp-GAL4,UAS-GFP/srp-GAL4,UAS-red stinger;repo*^*03702*^ (*repo*^*03702*^) and *w;srp-GAL4,UAS-GFP/srp-GAL4,UAS-red stinger;Df(3L)H99,repo*^*03702*^ (*Df(3L)H99,repo*^*03702*^).

Given the impaired migration of macrophages in *repo* mutant embryos, we analysed their inflammatory responses to sterile, laser-induced wounds. Wounding of *repo* mutant embryos revealed that reduced numbers of macrophages reached wound sites by 60-min post wounding compared with controls (Fig. 5a, b, d). Since there are fewer macrophages present locally at this developmental stage in *repo* mutants (Fig. 5c), the percentage of cells responding to the injury was also quantified, revealing a significant decrease in the ability of cells to respond to wounds (Fig. 5e; Supplementary Movie 1). Early migration away from wounds does not appear to underlie these defects (Fig. 5f), suggesting that excessive amounts of apoptotic cells turn off wound responses in *repo* mutants. To further evidence this conclusion, we removed developmental apoptosis, comparing wound responses in *Df(3L)H99* mutants and *Df(3L)H99,repo* double-mutant embryos. Wound responses are defective in *Df(3L)H99* mutants^32^, though we have found this phenotype to exhibit variable penetrance (Fig. 6; Roddie et al.^31^). Therefore, at best we would expect a rescue of the more severe *repo* mutant phenotype to *Df(3L)H99* levels, though in this instance we did not detect a strong *Df(3L)H99* defect in controls (Fig. 6e, f).

In contrast to the rescue of migration speed, removing apoptosis from a *repo* mutant background failed to rescue either the numbers of cells responding to the injury or the percentage of cells able to respond (Fig. 6). Therefore, the underlying defect in *repo* mutants that hinders macrophage inflammatory responses does not appear to be contact with apoptotic cells (Fig. 6), in contrast to the effect seen for migration defects (Fig. 4).

### Neurons and glia respond to injury through changes in their calcium dynamics

**Fig. 7:**
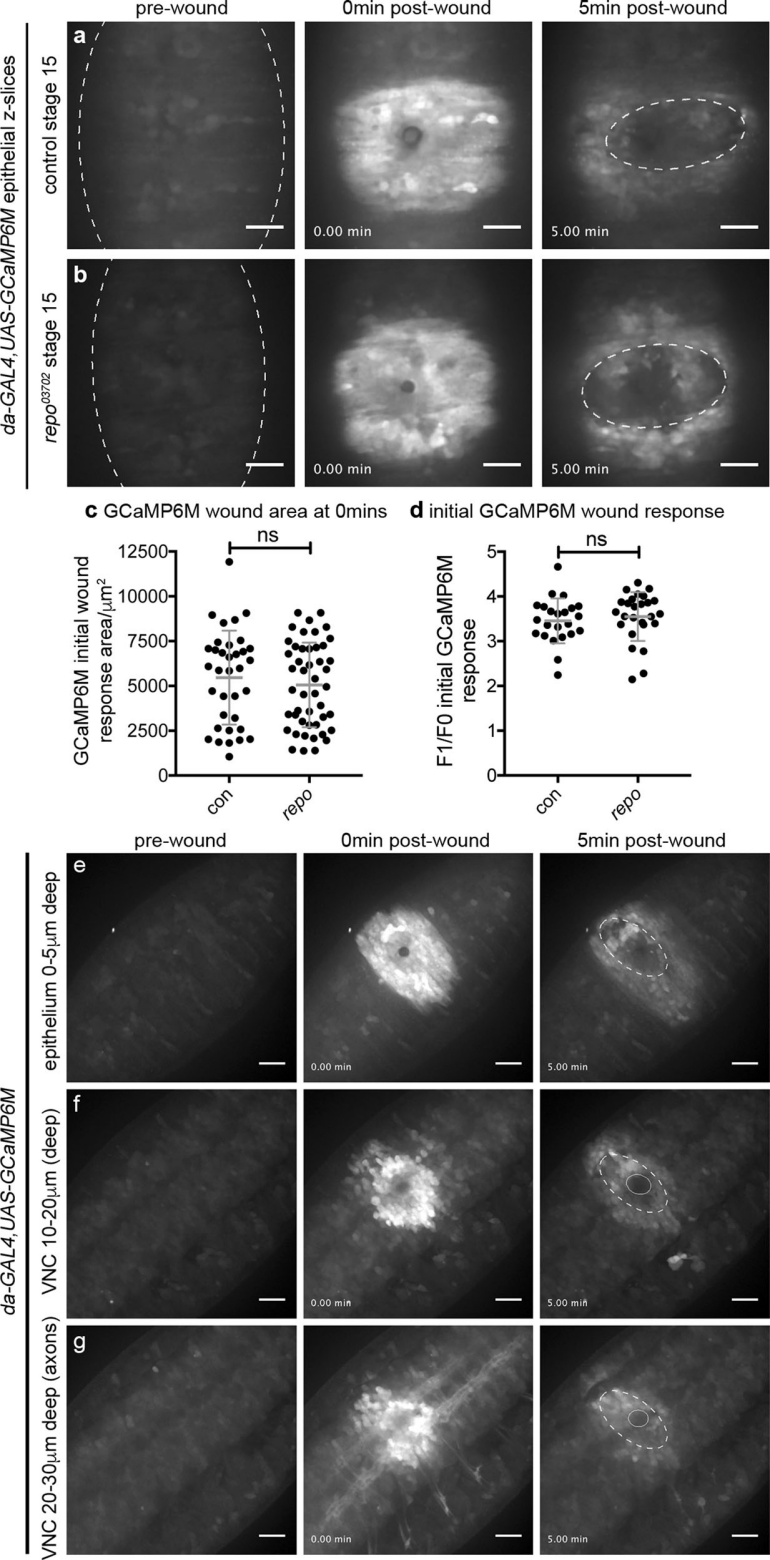
Defective calcium responses to wounding in *repo* mutants are restricted to less superficial tissues. **a**, **b** Calcium levels imaged via ubiquitous expression of GCaMP6M (*da-GAL4,UAS-GCaMP6M*) on wounding of the ventral surface of control (**a**) and *repo* mutant (**b**) stage 15 embryos; images show pre-wound calcium levels, immediately after wounding (0 min) and 5 min after wounding. Dotted lines show edges of embryo and wound edges in pre-wound and 5 min images, respectively. **c** Scattergraph showing area of GCaMP6M response immediately after wounding; Mann–Whitney test used to compare control vs *repo* (*n* = 35 and 47, respectively; *P* = 0.66). **d** Scattergraph showing ratio of GCaMP6M intensity in wound area before and immediately after wounding; Mann–Whitney test used to compare control vs *repo* (*n* = 23 and 26, respectively; *P* = 0.22). **e**–**g** maximum projections of superficial/epithelial regions (0–5 μm, **e**), superficial half of the VNC (10–20 μm from surface, **f**) and deeper/dorsal half of the VNC (20–30 μm from the surface, **g**) of the ventral side of a wounded control embryo containing ubiquitous expression of GCaMP6M; panels show pre-wound calcium levels, immediately after wounding (0 min) and 5 min after wounding. Dotted lines show edges of epithelial wound at 5 min, solid circles show physical damage at deeper regions of the embryo; embryo has not been orientated in order to show larger region of the response to wounding. Lines and error bars in scattergraphs show mean and standard deviation, respectively; scale bars represent 20 μm; ns indicates not significant (*P* > 0.05). Genotypes are *w;;da-GAL4,UAS-GCaMP6M* (control) and *w;;repo*^*03702*^*,da-GAL4,UAS-GCaMP6M* (*repo*).

**Fig. 8:**
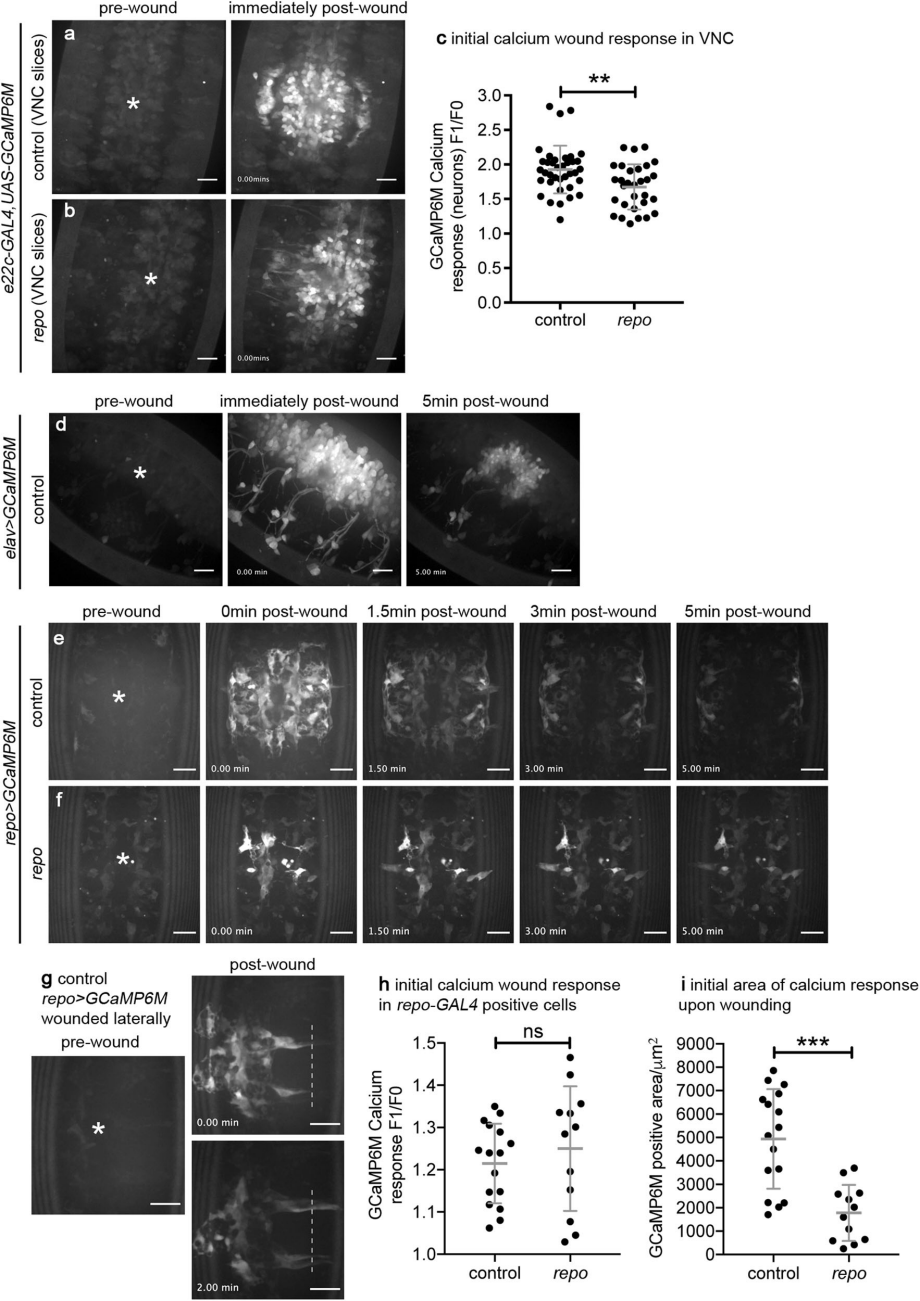
Non-epithelial tissues contribute to wound responses. **a**, **b** Calcium levels within the VNC imaged via expression of *UAS-GCaMP6M* via *e22c-GAL4* (projections assembled from slice range 30–10-μm deep from surface of the embryo) on wounding of the ventral surface of control (**a**) and *repo* mutant (**b**) stage 15 embryos; images show pre-wound calcium levels and immediately after wounding (0 min). **c** Scattergraph showing GCaMP6M response within VNC immediately after wounding (F1/F0) of control and *repo* mutants labelled using *e22c-GAL4,UAS-GCaMP6M*; Mann–Whitney test used to compare control vs *repo* (*n* = 37 and 30, respectively; *P* = 0.0051). **d** Calcium levels in neuronal cells (labelled using *elav-GAL4* to drive *UAS-GCaMP6M* expression) on wounding of the ventral surface of a stage 15 embryo; images show pre-wound calcium levels, immediately after wounding (0 min) and 5-min after wounding. **e**–**g** Calcium levels in glial cells (labelled using *repo-GAL4* to drive *UAS-GCaMP6M* expression) on wounding of the ventral surface of control (**e**, **g**) and *repo* (**f**) mutant stage 15 embryos; images show pre-wound calcium levels, immediately after wounding (0 min) and 5-min after wounding. **g** shows embryo wounded more laterally and subsequent spread of calcium signal along glial cells to more lateral positions; dotted line shows equivalent position in the 0 min and 2 min post-wound timepoint images. **h**, **i** Scattergraphs showing GCaMP6M responses in glial cells immediately after wounding (**h**, F1/F0) and the initial area (μm^2^) of the GCaMP6M response in control and *repo* mutant embryos labelled via *repo-GAL4,UAS-GCaMP6M* (**i**); Mann–Whitney test used to compare control vs *repo* (*n* = 16 and 12, respectively; *P* = 0.42 (**h**) and *P* = 0.0003 (**i**)). Asterisks show position of wounds in pre-wound images; scale bars represent 20 μm; lines and error bars show mean and standard deviation in all scattergraphs; **, *** and ns denote *P* < 0.01, *P* < 0.001 and not significant (*P* > 0.05), respectively. Genotypes are *w;e22c-GAL4,UAS-GCaMP6M* (**a**, **c**), *w;e22c-GAL4,UAS-GCaMP6M;repo*^*03702*^ (**b**, **c**), *elav-GAL4/w;UAS-GCaMP6M/+* (**d**), *w;repo-GAL4,UAS-GCaMP6M* (**e**, **g**, **h**–**i**) and *w;repo-GAL4,UAS-GCaMP6M;repo*^*03702*^ (**f**, **h**, **i**).

Since removing the apoptotic cell burden from macrophages in *repo* mutants failed to improve their inflammatory responses to wounds (Fig. 6), we investigated whether upstream signalling mechanisms that form part of the normal response to wounds remain intact in this mutant background. Laser ablation of embryos triggers the rapid spread of a calcium wave through the epithelium, away from the site of damage (Fig. 7a; Supplementary Movie 2; Razzell et al.^14^). This wave of calcium activates hydrogen peroxide production via DUOX, which is required for migration of macrophages to wound sites^14,45,54^. Imaging calcium dynamics after wounding using the cytoplasmic calcium sensor GCaMP6M^41^, we were unable to identify a difference immediately post wounding in the epithelial calcium responses of *repo* mutant embryos compared with controls (Fig. 7a–d; Supplementary Movie 2). However, in performing these experiments we noticed that the calcium response was not limited to the epithelium, with this signal visible deeper within the embryo, including within the neurons and glia of the VNC (Fig. 7e–g)—a structure located immediately underneath the epithelium on the ventral side of the embryo (Fig. 1a, b). These changes in intracellular calcium were not limited to the damaged tissue and extended away from the necrotic core of the wound (Fig. 7e–g; Supplementary Movie 3), with changes in calcium levels particularly striking within the axons of the CNS (Fig. 7g). Strikingly, quantification of calcium responses in sub-epithelial regions upon wounding showed a reduced calcium response in *repo* mutants compared with controls (Fig. 8a–c), suggesting that glial cells are responsive to injury and contribute to damage-induced signalling.

### *repo* is required for normal calcium responses to injury in the developing embryo

Using the tissue-specific drivers *elav-GAL4* (neurons) and *repo-GAL4* (glia; n.b. *repo-GAL4* has been shown to label residual glial cells in a *repo* null mutant background—this reporter does not require *repo* function for expression of *GAL4*)^27^ to express GCaMP6M confirmed that both neurons and glial cells within the VNC respond to injury by transiently increasing their cytosolic calcium levels (Fig. 8d, e; Supplementary Movies 4 and 5). Changes in cytoplasmic calcium are transmitted beyond the confines of the wound (Fig. 8d, g) and remain elevated at wound sites several hours post injury (Supplementary Fig. 3). These responses suggest that these cells communicate damage signals away from sites of tissue damage, potentially contributing to inflammatory recruitment of macrophages and regenerative processes. In addition, the transmission of calcium signals distal to the site of physical injury suggests that these responses are not simply limited to those cells damaged during the wounding process.

In *repo* mutants, while there is less glial proliferation, residual glial cells are present, but lack late glial markers^21,22^ and normal patterns of phagocytic receptor expression^25^. In contrast to the wild-type situation, wounding of *repo* mutants with glial-specific expression of GCaMP6M showed a reduction in the calcium response on injury (Fig. 8e, f, h, i). Taken together, these data indicate that, in contrast to our present understanding, tissues other than the epithelium undergo alterations in calcium signalling upon injury. Furthermore, the reduced calcium responses observed in *repo* mutants on wounding may therefore contribute to the reduced inflammatory responses undertaken by macrophages.

## Discussion

Clearance of apoptotic cells is associated with reprogramming of phagocytes such as macrophages towards anti-inflammatory states and is part of the process of resolution of inflammation^2^. Here, we show that by impairing glial specification using *repo* mutants, macrophages can be challenged with increased levels of apoptotic cell death in vivo. Challenging ‘wild-type’ macrophages in this way causes them to become engorged with phagocytosed apoptotic cells. The excessive amounts of apoptosis macrophages face in *repo* mutants appear associated with impaired macrophage developmental dispersal, slowed migration speeds and attenuated inflammatory responses. However, while macrophage migration speeds can be rescued by removing apoptosis from a *repo* mutant background, wound responses are not improved, suggesting interactions with apoptotic cells do not represent the primary cause of this phenotype. Instead, in contrast to our current understanding, tissues in addition to the wounded epithelium are wound-responsive, and glia and the neurons they support are likely to contribute to immune cell recruitment and repair mechanisms in *Drosophila*.

Macrophages in *repo* mutants become highly vacuolated with those vacuoles containing apoptotic cells. At early stages, much apoptotic cell death around the developing VNC corresponds to dying epidermal cells at segmental boundaries^55^, but by stage 15 most apoptosis is confined to the VNC^56^. How macrophages access apoptotic cells that *repo* mutant glia have failed to clear remains to be determined precisely. However, the blood–brain barrier (BBB), which is comprised of glial cells, is yet to form at the stages we have analysed^57^. Normally macrophages are excluded from the VNC, but can enter this structure in *repo* mutants at later stages^25^, suggesting the BBB fails to form in an appropriate manner. Interestingly, active migration of dying neurons has been observed in the zebrafish brain to aid their clearance by macrophages^58^, and apoptotic cells are expelled towards the periphery of the VNC during development^47^. Therefore, a similar process may occur in flies facilitating contact between macrophages and apoptotic cells in *repo* mutants. In the absence of macrophages, glial cells become engorged with apoptotic cells, highlighting the interplay and competition for apoptotic cells between these phagocytes^47^.

The vacuolated macrophages in *repo* mutants exhibit impaired dispersal and reduced migration speeds, phenotypes consistent with other *Drosophila* mutants that perturb apoptotic cell clearance, including *SCAR/WAVE*^51^ and *simu*^31^. Similarly, removal of the apoptotic clearance burden via ablation of the apoptotic machinery improves migration speeds in these mutant backgrounds. This suggests that apoptotic cells are responsible for these phenotypes and that the ‘migratory substrate' (i.e., the VNC) is sufficiently developed to support normal motility. Structural defects in the VNC are also unlikely to account for perturbed developmental dispersal of macrophages, since we and others have found that VNC defects are not apparent until after completion of macrophage dissemination^21,22^. Excessive production of find-me cues—chemoattractants released from apoptotic cells to alert phagocytes to their presence^59^—represents one potential explanation, with such cues distracting or overwhelming macrophages. Consistent with this, apoptotic cells have been inferred to be the most highly prioritised migratory cue for macrophages in the fly embryo^53^, and the degree of vacuolation does not correlate with decreased migration speeds in *repo* or *simu* mutants^31^, revealing that apoptotic cells need not be engulfed in order to affect migration speeds. Currently, this hypothesis remains difficult to investigate further, since the nature and identity of find-me cues have yet to be discovered in this organism. Alternatively, reprogramming of macrophages to different transcriptional and/or activation states may underlie these phenotypic changes^60,61^. While little evidence for such reprogramming exists in *Drosophila*, infection can drive *Drosophila* macrophage metabolism towards aerobic glycolysis^62^, resembling pro-inflammatory activation of vertebrate myeloid cells. Another recent paper used single-cell RNA-sequencing to identify subsets of larval blood cells with molecular signatures reminiscent of immune cell activation^63^. Therefore, emerging data suggest that *Drosophila* macrophages are more similar to their vertebrate counterparts than previously anticipated and reprogramming via apoptotic cell clearance remains a viable explanation for the changes in their behaviour in *repo* mutants.

Phagocytes such as *Drosophila* macrophages may have to decide whether to engulf or move: for instance both dendritic cells and *Dictyostelium* amoebae pause during macropinocytosis^64,65^, a process related to phagocytosis. Similarly, lysosomal storage disorders that lead to vacuolation are associated with perturbed immune cell migration in patient-derived cells^66–68^ and experimental models^69^. Sequestration of regulators of both phagocytosis and motility, such as the lysosomal Trp channel Trpml (the fly homologue of TRPML1/MCOLN1)^70^, by excessive phagocytic cup formation in the face of elevated apoptotic cell burdens, could antagonise the ability of phagocytes to migrate. *repo* mutants represent a model to further understand how apoptotic cells regulate changes in macrophage behaviour in vivo, such as their migration and phagocytic capacities, especially since initial specification of macrophages does not appear compromised in this background and *repo* plays no direct functional role in these cells.

Macrophage inflammatory responses were not improved by blocking apoptosis in *repo* mutants. This was surprising, since preventing apoptosis in *simu* mutants, another background in which macrophages face large numbers of apoptotic cells, did improve macrophage responses to injury^31^. Thus it is unlikely that apoptotic cell–macrophage interactions represent the primary cause of wound recruitment defects in *repo* mutants. Faster and more sensitive imaging technologies alongside the use of alternative drivers to express genetically encoded calcium reporters enabled capture of larger volumes of the embryo during wounding in comparison to previous studies^14^. This revealed that those tissues surrounding the damaged epithelium, such as the neurons and glia of the VNC, were also responsive to injury. Loss of *repo* function causes defects in glial specification, proliferation^21–23^ and also leads to a perturbed calcium response in the VNC and glia. Potentially, *repo* drives a transcriptional programme enabling glia to respond to injury. Failed glial specification may have a ‘knock-on' effect and hinder the damage responses of neurons, which are supported by glial cells. Alternatively, the decreased number or mispositioning of glial cells in *repo* mutants^21^ may decrease the amplitude or spread of wound signals activated by injury, such as via defective cell–cell contacts between glia. Impairing the ability of surrounding tissues to respond to injury may therefore perturb the generation of wound cues required for normal macrophage migration to sites of tissue damage and could potentially impact evolutionarily conserved CNS repair processes^71^, especially since calcium waves in the developing *Drosophila* wing disc are required for regeneration following mechanical injury^72^. While these results mean that *repo* mutants are less suitable to study apoptotic cell–macrophage interactions after injury, this work has changed our understanding of how wound responses are regulated in *Drosophila*.

The use of calcium as a regulator of wound responses is conserved across evolution with calcium waves visible upon transection of zebrafish larval fins^17^, and immediately after wounding *Xenopus* embryos^73^ and the *C. elegans* epidermis^74^. Release of calcium from internal stores during wave propagation is also a commonality in these models. The mechanism of activation in the *Drosophila* CNS remains to be established, but in zebrafish tailfin wounds the release of ATP from damaged cells may activate P2Y receptors, leading to subsequent release of calcium from internal stores via PKC signalling^75^. Calcium waves can also be observed within the developing zebrafish brain upon wounding, with these dependent upon glutamate-mediated activation of NMDA receptors. NMDA receptors in turn regulate ATP-dependent recruitment of microglial cells to sites of injury^76^. Traumatic brain injury has long been associated with rapid decreases in extracellular calcium (e.g., Young et al.^77^; for review see Weber^78^) and laser wounding has been proposed as a useful technique to model this^79^. Indeed, buffering calcium dynamics within damaged neurons in flies can be neuroprotective^80^, suggesting this system could uncover novel therapeutic strategies for traumatic brain injury.

In summary, we have shown that increasing the apoptotic cell burden placed on macrophages impairs their normal behaviour, dampening their migratory abilities and impairing their dispersal; *repo* mutants will thus provide a useful model to understand the numerous functions of macrophages relating to contact with apoptotic cells that do not depend upon intact glial function. Furthermore, we have uncovered that wound-induced calcium waves spread beyond the epithelium into neighbouring tissues, potentially enabling them to contribute to recruitment of macrophages and subsequent repair processes. How these waves spread and how epithelial and non-epithelial tissues integrate these responses to coordinate inflammation and repair remain key questions for future work. Since specification of macrophages is not perturbed in *repo* mutants, this model will prove useful to examine macrophage–apoptotic cell interactions in vivo in more detail and shed light on cellular interactions that are fundamental for normal development and homeostasis.

## Supplementary information

Supplementary Information

Supplementary Figure 1

Supplementary Figure 2

Supplementary Figure 3

Supplementary Table 1

Supplementary Table 2

Supplementary Movie 1

Supplementary Movie 2

Supplementary Movie 3

Supplementary Movie 4

Supplementary Movie 5

## Data Availability

Supplementary information is available at Cell Death and Disease’s website. Fly lines and raw data are available on request from I.R.E.
