# Supplementary material for: Overexposure to apoptosis via disrupted glial specification perturbs Drosophila macrophage function and reveals roles of the CNS during injury
Source: Cell Death Dis. 2020 Aug 14;11(8):627. doi: 10.1038/s41419-020-02875-2 (PMC7428013; doi:10.1038/s41419-020-02875-2)
Supplement: Supplementary file 1 — Supplementary Information [file 41419_2020_2875_MOESM1_ESM.docx]

# Supplementary information

# Supplementary Figure, Table and Movie Legends

## Supplementary Figure 1. VNC defects are apparent at late but not early stages of development in *repo* mutants

(a) schematic showing measurement of the anterior-posterior length of the 5 most-central body segments on the ventral side of the embryo. (b-c) scattergraphs showing the anterior-posterior length of the 5 most-central embryonic body segments at stage 15 (b) and stage 13 (c) in *repo* mutants and control embryos; lines and error bars show mean and standard deviation, respectively; n=12 and 8 (b) and 13 and 13 (c) control and *repo* mutant embryos, respectively; values are shown normalised to the control average at each stage; statistical analysis via Mann-Whitney tests.

## Supplementary Figure 2. *repo* mutants contain the same number of macrophages as control embryos

(a-b) flat preparations of stage 15 control (a) and *repo* mutant (b) embryos containing red stinger-labelled macrophages (via *crq-GAL4,UAS-red stinger*). Multiple fields of view combined to represent entire embryo using MosaicJ in Fiji; anterior is left; scale bars represent 100μm. (c) scattergraph of numbers of cells per embryo showing no difference between controls and *repo* mutants (p=0.46 via Mann-Whitney test; n=10 and 13). Lines and error bars represent mean and standard deviation, respectively; ns denotes not significant. See Supplementary Methods section below for details of this analysis.

## Supplementary Figure 3. Maintenance of elevated calcium levels at wound sites

(a-b) *w^1118^;;da-GAL4,UAS-GCaMP6M* embryos 1 hour (a) and 2 hours (b) post-wounding showing elevated calcium levels at wound sites. Scale bars represent 100μm.

## Supplementary Table 1. University of Sheffield fly food ingredients and suppliers

## Supplementary Table 2. Genotypes and sources of alleles used in this study

## Supplementary Movie 1. Defective inflammatory responses to injury in the absence of *repo* function

Response of GFP and red stinger-labelled macrophages to injury in control and *repo* mutant embryos. Embryos wounded on the ventral midline at stage 15. Scale bar represents 20μm. Genotypes are *w^1118^;srp-GAL4,UAS-red stinger/srp-GAL4,UAS-GFP* (control) and *w^1118^;srp-GAL4,UAS-red stinger/srp-GAL4,UAS-GFP;P{PZ}repo^03702^* (*repo*).

## Supplementary Movie 2. Epithelial calcium responses to injury in stage 15 control and *repo* mutant embryos

Movies of epithelial cell calcium responses to injury at stage 15 in control and *repo* mutant embryos. Calcium imaged via *w^1118^;;da-GAL4,UAS-GCaMP6M* and projections assembled from superficial slices of z-stacks. Embryos wounded on the ventral midline. Scale bars show 20μm. Movies correspond to stills shown in Figure 7a-b.

## Supplementary Movie 3. Calcium responses of non-epithelial tissue following wounding

Movie of calcium responses to injury at stage 15 at different depths within the embryo. Calcium dynamics visualised via ubiquitous expression of GCaMP6M (*w^1118^;;da-GAL4,UAS-GCaMP6M*) following wounding on the ventral midline. Movie assembled from slices corresponding to superficial z-slices of the image stack (epithelial cells, 0-5μm from the surface of the embryo; top panel), slices of the ventral half of the VNC (10-20μm from the surface of the embryo; central panel) and the dorsal half of the VNC (20-30μm from the surface of the embryo; bottom panel). Scale bar shows 20μm. Movie corresponds to stills shown in Figure 7e-g.

## Supplementary Movie 4. Neuronal calcium responses to injury

Movie of neuronal calcium response to injury at stage 15. Calcium imaged in neurons via *elav-GAL4/w[1118];UAS-GCaMP6M/+*. Embryo wounded laterally with respect to the ventral midline. Scale bar shows 20μm. Movie corresponds to stills shown in Figure 8d.

## Supplementary Movie 5. Calcium responses of glial cells to injury in control and *repo* mutant embryos

Movies of glial calcium responses to injury at stage 15 in control and *repo* mutant embryos. Calcium imaged via *repo-GAL4,UAS-GCaMP6M*. Embryos wounded on the ventral midline. Scale bars show 20μm. Movies correspond to stills shown in Figure 8e-f. Genotypes are *w^1118^;repo-GAL4,UAS-GCaMP6M* (control) and *w^1118^;repo-GAL4,UAS-GCaMP6M;P{PZ}repo^03702^* (*repo*).

# Supplementary methods

## Flat preparation of embryos for whole embryo macrophage counts

Embryos containing *crq-GAL4,UAS-red stinger* to label macrophage nuclei were fixed as per normal immunostaining protocols (see Methods section). Fixed embryos were then washed in PBS and transferred to DABCO mountant (25 mg/ml dissolved in 1X PBS/90% w/v glycerol) and left overnight at room temperature. Embryos were then gently compressed under a coverslip (thickness 1.5) to flatten embryos; multiple fields of view were then taken using a Leica MZ205 fully automated fluorescent stereomicroscope and assembled into a montage using the MosaicJ plugin in Fiji. These images were then thresholded to create binary images in Fiji before cell numbers were quantified automatically using the analyse particles tool in Fiji.
