# Supplementary figures and images for: Overexposure to apoptosis via disrupted glial specification perturbs Drosophila macrophage function and reveals roles of the CNS during injury
Source: Cell Death Dis. 2020 Aug 14;11(8):627. doi: 10.1038/s41419-020-02875-2 (PMC7428013; doi:10.1038/s41419-020-02875-2)

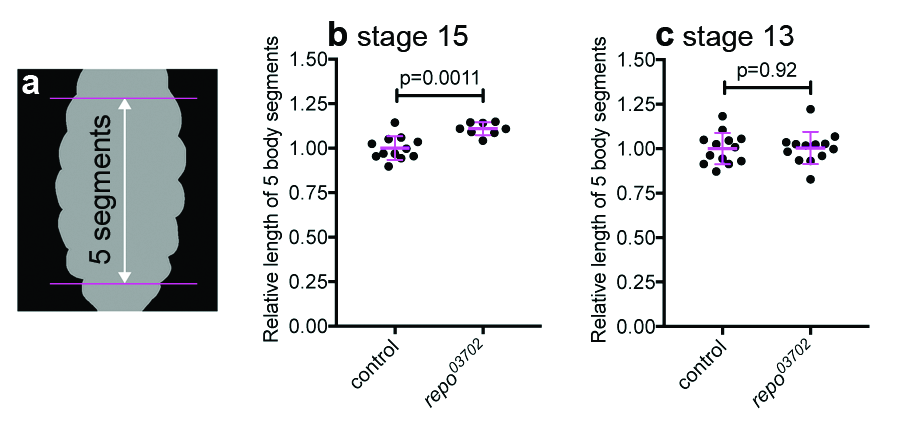

Supplement: Supplementary file 2 — Supplementary Figure 1 [file 41419_2020_2875_MOESM2_ESM.tif]

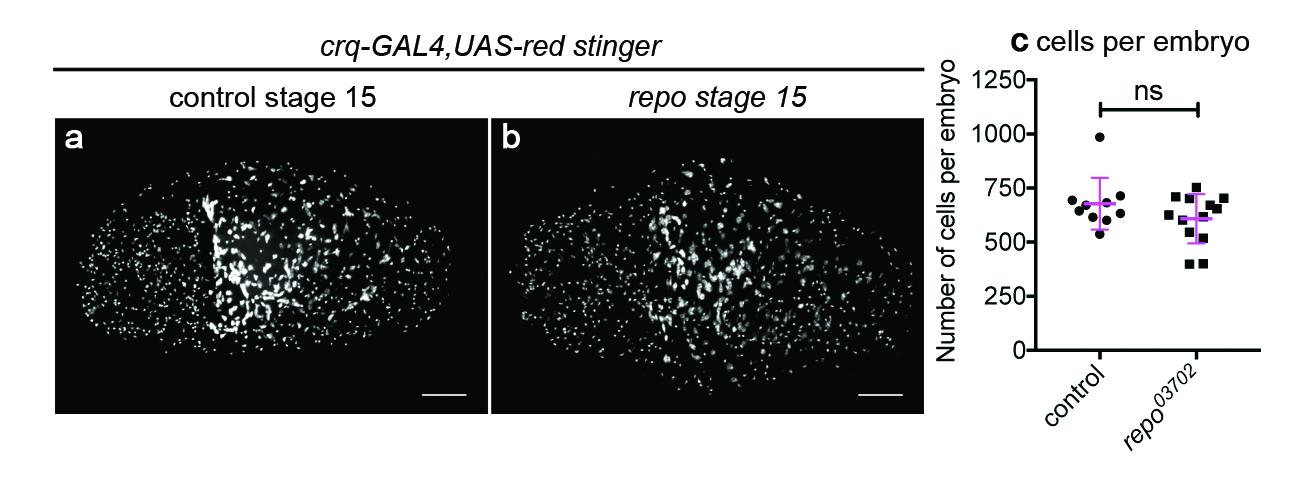

Supplement: Supplementary file 3 — Supplementary Figure 2 [file 41419_2020_2875_MOESM3_ESM.tif]

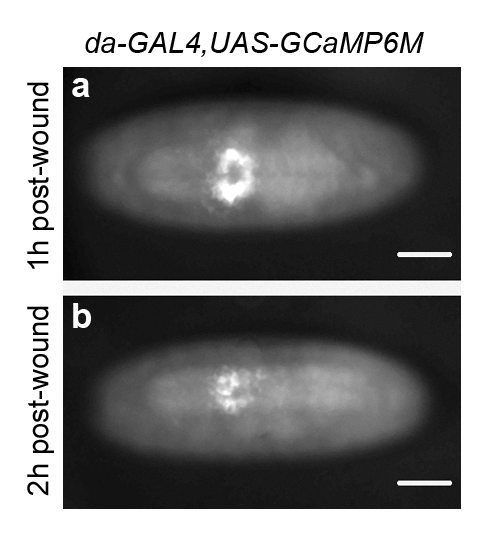

Supplement: Supplementary file 4 — Supplementary Figure 3 [file 41419_2020_2875_MOESM4_ESM.tif]
